# Glucocorticoid nanoformulations relieve chronic pelvic pain syndrome and may alleviate depression in mice

**DOI:** 10.1186/s12951-023-01893-4

**Published:** 2023-06-21

**Authors:** Yang Yang, Ruimin Hu, Jun Zheng, Qianmei Wang, Senlin Xu, Zhansong Zhou, Dinglin Zhang, Wenhao Shen

**Affiliations:** 1 Department of Urology, Southwest Hospital, Army Medical University, Third Military Medical University, Chongqing, 400038 China; 2 Department of Chemistry, College of Basic Medicine, Army Medical University, Third Military Medical University, Chongqing, 400038 China; 3 Department of Pharmacy, Southwest Hospital, Army Medical University, Third Military Medical University, Chongqing, 400038 China; 4 Department of Pathology, Southwest Hospital, Army Medical University, Third Military Medical University, Chongqing, 400038 China

**Keywords:** Chronic pelvic pain syndrome, Dexamethasone, Nanomedicines, Targeted therapy, Smart drug release

## Abstract

**Background:**

Chronic pelvic pain syndrome (CPPS) is a typical symptom of chronic prostatitis (CP) in males that may cause abnormal urination, sexual dysfunction, or depression and significantly affect the quality of life of the patient. Currently, there is no effective treatment for CPPS due to its recurrence and intractability. For synergistic CPPS therapy, we developed pH/reactive oxygen species (ROS) dual-responsive dexamethasone (Dex) nanoformulations using a ROS-responsive moiety and phytochemical modified α-cyclodextrin (α-CD) as the carrier.

**Results:**

Dex release from the nanoformulations can be controlled in acidic and/or ROS-rich microenvironments. The fabricated Dex nanoformulations can also be efficiently internalized by lipopolysaccharide (LPS)-stimulated macrophages, prostatic epithelial cells, and stromal cells. Moreover, the levels of proinflammatory factors (e.g., TNF-α, IL-1β, and IL-17 A) in these cells were significantly decreased by Dex nanoformulations treatment through the release of Dex, phytochemical and elimination of ROS. In vivo experiments demonstrated notable accumulation of the Dex nanoformulations in prostate tissue to alleviate the symptoms of CPPS through the downregulation of proinflammatory factors. Interestingly, depression in mice may be relieved due to alleviation of their pelvic pain.

**Conclusion:**

We fabricated Dex nanoformulations for the effective management of CPPS and alleviation of depression in mice.

**Supplementary Information:**

The online version contains supplementary material available at 10.1186/s12951-023-01893-4.

## Background

Chronic prostatitis (CP) is a common urological condition that affects approximately 8.2% of men worldwide [1]. CP can cause pain and discomfort in the pelvic region, abnormal urination and sexual dysfunction, and even dizziness, headache, and depression [2]. The symptoms are characterized by periodic exacerbations followed by tolerable symptoms, a clinical process that is constantly changing and seriously affects the patient’s quality of life [2]. In recent years, several studies have shown that CP may also lead to the occurrence of prostate cancer [3–5]. In 1995, the National Institutes of Health (NIH) classified prostatitis into four categories: Type I and II prostatitis are related to acute and chronic bacterial infection, respectively; Type III prostatitis, which is also called chronic pelvic pain syndrome (CPPS); and Type IV prostatitis, which does not show any clinical symptoms and is found only by accident [6]. Type I and IV prostatitis account for low proportions of the incidence of prostatitis and are easy to treat. Type II and III prostatitis account for approximately 5-8% and 90% or more of the cases of prostatitis, respectively [6]. Consequently, researchers have mainly focused on overcoming the barriers of Type II and III prostatitis treatment. Antibiotics have been used clinically to treat Type II prostatitis [7]. The main obstacle with this treatment is that antibiotics have difficulty penetrating the prostatic epithelium to kill the interior bacteria in the prostatic lumen. Our previous work developed reactive oxygen species (ROS)-responsive nanoparticles encapsulating cefpodoxime proxetil for targeted treatment of Type II prostatitis [8]. This nanodrug delivery system can effectively penetrate the prostate epithelial barrier and deliver antibiotics into the prostate lumen to eradicate bacteria.

Although the pathogenesis of Type III prostatitis (CPPS) remains largely unknown, bacterial infection, urinary reflux, autoimmune responses, neuroendocrine disorders, and anxiety-related factors have been reported to be responsible [9]. The 2022 European Urology Guidelines noted that alleviation of the clinical symptoms of Type III prostatitis is the main goal for the treatment of this disease. Anti-inflammatory drugs are the first choice for relieving the symptoms of Type III prostatitis, which is primarily prostate pain [6]. Compared to a placebo, anti-inflammatory drugs were found to be effective in 80% of CPPS patients [10]. Both steroidal anti-inflammatory drugs (e.g., dexamethasone (Dex) and prednisolone) and nonsteroidal anti-inflammatory drugs (e.g., rofecoxib and celecoxib) are employed in the treatment of CPPS [2]. Although steroidal anti-inflammatory drugs show good therapeutic efficacy for the treatment of CP [11], their side effects after long-term administration cannot be ignored. In addition, the effectiveness of drugs for the treatment of CPPS needs to be further improved. Given the recurrence and intractability of CPPS, a new strategy for the effective management of CPPS is urgently needed.

In recent years, nanotherapeutics have been widely developed for the diagnosis and treatment of inflammatory diseases ^[12]–[13]^. Nanoformulations can deliver therapeutics (e.g., drugs, anti-inflammatory mediators, anti-inflammatory peptides, and genes) or modulate the inflammatory microenvironment to manage inflammatory diseases ^[12, 14]–[15]^. For example, excessive ROS production in biological systems can cause oxidative stress, which is closely related to inflammatory diseases; therefore, scavenging ROS by nanotherapeutics has been widely employed for the treatment of such diseases ^[16]–[17]^. In addition, the low pH values and high concentrations of ROS in inflammatory sites are beneficial for the smart release of therapeutics from the nanoformulations [18–21]. Although great success has been achieved with the use of nanotherapeutics for the treatment of inflammatory diseases, few studies have focused on CP therapy. Our previous work demonstrated that inflammatory prostate tissue is infiltrated by a large number of activated macrophages. Moreover, the high concentrations of ROS and overexpression of folate receptors that have been detected in these cells provide an appropriate microenvironment for targeted and smart drug delivery for the treatment of CP [8]. In addition, the controlled release of therapeutics in CP may be achieved due to the slightly acidic microenvironments of endosomes (pH 5–6) and lysosomes (pH 4–5) in macrophages [22].

**Fig. 1:**
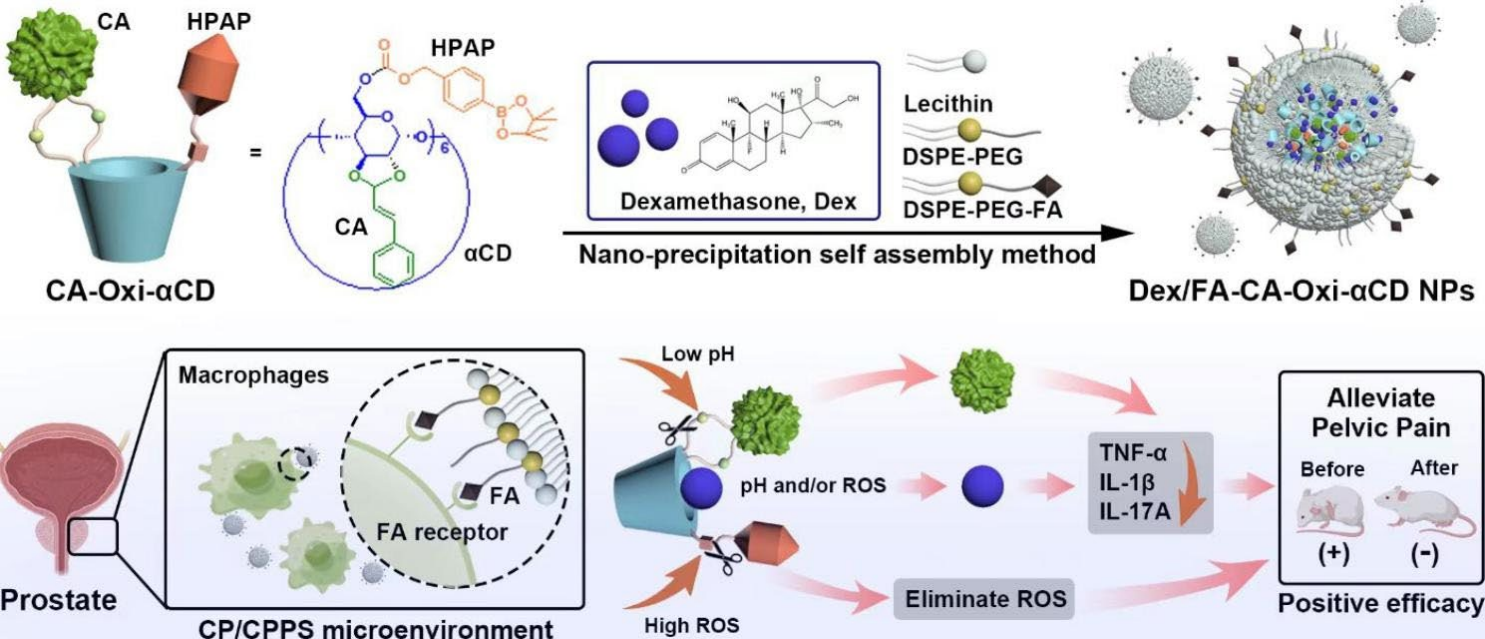
Fabrication of Dex-loaded pH/ROS dual-responsive NPs for targeted chronic prostatitis treatment

As mentioned above, our previous work investigated the efficacy and mechanism of nanomedicines for the targeted treatment of Type II prostatitis. To synergistically treat CPPS and thoroughly release therapeutics in inflammatory prostate tissue, herein, we designed a three-in-one multifunctional nanomedicine for combating CPPS (Fig. 1). The pH/ROS dual-responsive carrier was synthesized by modifying cyclodextrin (CD) with cinnamaldehyde (CA, a phytochemical with anti-inflammatory activities) [23] and 4-(hydroxymethyl) phenylboronic acid pinacol ester (HPAP, a ROS scavenger) [24] (Figure S1). Using the synthesized carrier, Dex-loaded, folic acid (FA)-modified nanoparticles (named Dex/FA-CA-Oxi-αCD NPs) were fabricated via a nanoprecipitation self-assembly method (Fig. 1). In vitro experiments demonstrated that Dex/FA-CA-Oxi-αCD NPs can be internalized by inflammatory cells in prostate tissue, and the internalized NPs can be degraded in a low pH and/or ROS-rich microenvironment. In vivo experiments verified that the Dex nanoformulations can effectively accumulate in prostate tissue and significantly alleviate pelvic pain through the elimination of ROS or downregulation of proinflammatory factors such as TNF-α, IL-1β, and IL-17 A. Interestingly, our results demonstrated that these NPs may alleviate depression in mice by relieving their pelvic pain. Our results provide a practicable strategy for the targeted treatment of CPPS.

## Materials and methods

### Reagents

Lecithin was provided by Alfa Aesar (Shanghai, China). HPAP, 4-dimethyl aminopyridine (DMAP), 1-(3-dimethylaminopropyl)-3-ethyl carbodiimide hydrochloride (EDC•HCl), 1,1′-carbonyldiimidazole (CDI), poly (lactic-co-glycolic acid) (PLGA) and Pluronic F-127 (polyethylene-polypropylene glycol) were purchased from Sigma‒Aldrich Co. (Shanghai, China). CA and α-cyclodextrin (α-CD) were provided by Tokyo Chemical Industry Co., Ltd. (Tokyo, Japan). 1,2-Distearoyl-sn-glycero-3-phosphoethanolamine-N-methoxy (polyethylene glycol)-2000 (DSPE-PEG_2000_) and folic acid (FA)-conjugated 1,2-distearoyl-sn-glycero-3-phosphoethanolamine-N-methoxy (polyethylene glycol)-3400 (DSPE-PEG_3400_-FA) were supplied by Xi’an Ruixi Corporation (Xi’an, China). Cy5 free acid and Cy5-NHS ester were purchased from Lumiprobe, LLC. (Hallandale Beach, FL, USA). Dulbecco’s modified Eagle’s medium (DMEM) and fetal bovine serum (FBS) were obtained from HyClone Inc. (Waltham, MA, USA). Streptomycin–penicillin solution was purchased from Solarbio Life Sciences Co., Ltd. (Beijing, China). Antifade mounting medium with DAPI, Cell Counting Kit-8 (CCK-8), a Hydrogen Peroxide Assay Kit, an Annexin V-FITC Apoptosis Detection Kit, lipopolysaccharide (LPS), and LysoTracker Green were provided by Beyotime Biotechnology Co., Ltd. (Shanghai, China). Methyl alcohol and dimethyl sulfoxide (DMSO) were provided by Kelong Chemical (Chengdu, China). Dex was purchased from MedChemExpress (Shanghai, China). Antibodies against TNF-α, IL-17 A, and IL-1β and ELISA kits for TNF-α, IL-17 A, and IL-1β were purchased from Bio-Techne China Co., Ltd. (Shanghai, China). Antibodies against the 5-HT1A receptor, serotonin transporter (SERT), and glial fibrillary acidic protein (GFAP) were purchased from Affinity Biosciences (Suzhou, China). Cy5-conjugated Oxi-αCD was fabricated using our previously reported methods ^[25]–[26]^. Methods for the synthesis of the CA-Oxi-αCD materials are listed in the supporting information (Scheme S2-3).

### Cells and animals

Mouse monocyte macrophage RAW264.7 cells were purchased from the Cell Bank of the Chinese Academy of Sciences (Shanghai, China). Bluefbio Biotechnology Development Co., Ltd. (Shanghai, China) provided mouse prostatic epithelial cells and human prostatic stromal cells. Cells were cultured in DMEM with 10% FBS, 100 U/mL penicillin, and 100 µg/mL streptomycin, plated in a 25 mL cell culture flask, and cultured in a 37 °C incubator supplemented with 5% CO_2_.

Adult male NOD/SCID mice (6–8 weeks old) were provided by Hunan Sta Laboratory Animal Co., Ltd. (Changsha, China). All animals were housed in an SPF-grade sterile animal room. The room temperature was 22–25 °C and the relative humidity was 50-60% with a light-dark cycle of 12:12 h. Mice had free access to food and water. All animal experiments were conducted following guidelines approved by the Army Medical University Ethics Committee (Chongqing, China).

### Preparation and characterization of Dex-loaded NPs

Blank CA-Oxi-αCD NPs (blank NPs), Dex/CA-Oxi-αCD NPs, and FA-modified Dex/CA-Oxi-αCD NPs (Dex/FA-CA-Oxi-αCD NPs) were fabricated by a nanoprecipitation/self-assembly method as we previously reported [25]. Briefly, 6 mg of lecithin and 6 mg of DSPE-PEG_2000_ were dispersed in 400 µL of anhydrous ethanol and 7 mL of ultrapure water. Then, the solution was heated to 65 °C for 30 min. Additionally, 50 mg of CA-Oxi-αCD and 10 mg of Dex were dissolved in 400 µL of methyl alcohol and 200 µL of DMSO, and this mixture was added dropwise to the solution above. The resulting mixture was vortexed for 3 min, cooled to room temperature and allowed to self-assemble for 2 h. The suspension was centrifuged at 15,000 rpm for 10 min, and the collected precipitate was washed twice with 5% Pluronic F-127 and ultrapure water. Finally, the Dex/CA-Oxi-αCD NPs were collected. Subsequently, the solidified NPs were resuspended in 200 µL of ultrapure water. Dex/FA-CA-Oxi-αCD NPs were fabricated in the same way, except that 3 mg of DSPE-PEG_2000_ and 3 mg of DSPE-PEG_3400_-FA were used. Additionally, Cy5-conjugated Oxi-αCD was used to prepare Cy5-labeled NPs. An emulsion solvent evaporation method was used to fabricate the PLGA NPs. In brief, 50 mg of PLGA and 5 mg of Dex were dissolved in 0.7 mL dichloromethane, then 7.0 mL of 1% PVA was added, followed by sonication for 2 min sonication with a probe sonicator. Then the emulsion was poured into 0.3% PVA (20.0 mL) and stirred for 2 h at 25 °C. PLGA NPs were harvested by centrifugation at 10,000 rpm (7620 g).

Malvern Zetasizer Nano ZS equipment (Malvern, U.K.) was used to detect the NPs particle size, polydispersity index (PDI), and zeta potential. Transmission electron microscopy (TEM) (JEM-1400, Japan) was employed to observe the shape of the NPs. To determine the content of Dex in the NPs, 20 µL of Dex/CA-Oxi-αCD NPs or Dex/FA-CA-Oxi-αCD NPs were freeze-dried, weighed, dissolved in 1 mL of methanol and analyzed by high-performance liquid chromatography (HPLC). The drug loading (DL) of Dex in the NPs was calculated by the following formula:

DL% = (Dex content in NPs/NPs weight) × 100%.

### Cytotoxicity of the Dex-loaded NPs

Mouse macrophages, mouse prostatic epithelial cells, and human prostatic stromal cells were seeded in 96-well plates at a density of 1 × 10^4^ cells per well, and 100 µL of complete medium was added to each well for 24 h of incubation at 37 °C with 5% CO_2_. Then, the cells were treated with different concentrations of free Dex, blank NPs, Dex/CA-Oxi-αCD NPs, or Dex/FA-CA-Oxi-αCD NPs and cultured for another 24 h. Next, the medium was removed, the cells were washed with PBS 3 times, 100 µL of CCK-8 working solution (CCK-8:DMEM = 1:9) was added, and the cultures were incubated at 37 °C for 0.5 h. The absorbance at 450 nm was then measured by an Enzyme Standard Instrument (Thermo Varioskan Flash, USA).

### Intra- and extracellular H_2_O_2_ detection

Mouse macrophages, mouse prostatic epithelial cells, and human prostatic stromal cells were seeded in 12-well plates at a density of 2 × 10^5^ cells per well, incubated for 24 h, stimulated with LPS (1 µg/mL) for 12 h, washed 3 times with 1 mL of PBS, and then added to blank PLGA NPs, blank CA-Oxi-αCD NPs, Dex/CA-Oxi-αCD NPs or Dex/FA-CA-Oxi-αCD NPs. Then, the intra- and extracellular H_2_O_2_ concentrations were determined with a hydrogen peroxide assay kit.

### Cell apoptosis detection

Mouse prostatic epithelial cells and human prostatic cells were seeded in 6-well plates at a density of 2 × 10^5^ cells per well, stimulated with LPS (1 µg/mL) for 12 h. Free Dex, Dex/CA-Oxi-αCD NPs, or Dex/FA-CA-Oxi-αCD NPs were added for 24 h of incubation. Cells were then collected and resuspended in 500 µL of PBS. Next, 5 µL of FITC-annexin and 5 µL of propidium iodide (PI) (from the apoptosis assay kit) were added to the suspension. The apoptosis rate was determined by flow cytometry (BD FACS Aria III, USA).

### Cellular uptake

Mouse macrophages, mouse prostatic epithelial cells, and human prostatic stromal cells were seeded at a density of 1 × 10^5^ cells per well in confocal culture dishes for 24 h. Cells were stimulated with LPS (1 µg/mL) for 12 h and then coincubated with free Cy5, Cy5-labeled Dex/CA-Oxi-αCD NPs or Cy5-labeled Dex/FA-CA-Oxi-αCD NPs for 2 and 4 h (the fluorescence intensity of Cy5 in the drug was measured to be 1 µg/mL by the Enzyme Standard Instrument). Each dish was rinsed 3 times with PBS, and 1 mL of lysosome staining working solution (1 µL of LysoTracker/20 mL of DMEM) was added to each dish for incubation at 37 °C for 30 min. Subsequently, the cells were washed 3 times with PBS and fixed with 4% paraformaldehyde for 15 min. Finally, 100 µL of anti-fluorescence quenching mounting fluid containing DAPI was used to mount the cells. Confocal laser scanning microscopy (CLSM; LSM780, ZEISS, Germany) was used to observe cellular uptake ability.

### Detection of inflammatory factors in cells

Mouse macrophages and mouse prostatic epithelial cells were seeded in 6-well plates at a density of 2 × 10^5^ cells per well for 24 h and then stimulated with LPS (1 µg/mL) for 12 h. Saline (blank control), free Dex, Dex/CA-Oxi-αCD NPs, or Dex/FA-CA-Oxi-αCD NPs were added to each well for 24 h, and then western blot (WB) analysis and real-time fluorescence quantitative PCR (RT–qPCR) were used to determine the expression levels of the inflammatory factors TNF-α, IL-1β, and IL-17 A. Each experiment was repeated at least 3 times.

### Establishment of a murine model of experimental autoimmune prostatitis (EAP)

Rat prostatic protein extract (RPE) was collected as follows. The prostate tissue of 10 SD rats was stripped under sterile conditions, washed with normal saline, and cut into pieces. Then, 0.5% Triton X-100 and protease inhibitors were added, and the mixture was homogenized on ice in 0.01 M PBS (pH = 7.2). The solution was centrifuged at 10,000 × g for 30 min, and then the supernatant was aspirated. The protein concentration in the supernatant was detected using a BCA protein concentration assay kit, adjusted to 10 mg/mL with PBS, and stored frozen at -80 °C.

The establishment of the EAP mouse model was performed as reported in the literature [27]. In brief, after thawing at room temperature, RPE and Freund’s complete adjuvant (CFA) were mixed at a ratio of 1:1 and sonicated to make an emulsion. Male 6- to 8-week-old NOD/SCID mice were divided into model and control groups. Each mouse in the model group was subcutaneously injected with 150 µL of the above emulsion (25 µL into the right footpad, 25 µL into the left footpad, 50 µL into the base of the tail, and 50 µL into the shoulder). At the same time, each mouse was intraperitoneally injected with 1 ng of pertussis toxin (200 µL). Each mouse in the control group was subcutaneously injected with 150 µL of PBS at multiple points. The above antigens were injected again on the 15th day.

### In vivo biodistribution of Cy5-labeled NPs

Dex-loaded NPs were prepared and labeled with the fluorescent probe Cy5. EAP mice were divided into 4 groups with 5 mice in each group. Saline, free Cy5, Cy5-labeled Dex/CA-Oxi-αCD NPs, or Cy5-labeled Dex/FA-CA-Oxi-αCD NPs were intravenously injected into each EAP mouse via the tail vein. The fluorescence intensity of Cy5 was 20 µg per mouse, which was measured by an Enzyme Standard Instrument. In vivo fluorescence imaging was performed at 2, 4, 8, 24, and 48 h after injection using a Live Imaging System IVIS Spectrum CT (PerkinElmer, Waltham, USA) with an excitation filter at 625 nm and emission filter at 680 nm; mice were then sacrificed at 24 and 48 h. The heart, liver, spleen, lung, kidney, prostate tissue, and blood of the mice were collected, and the fluorescence intensity of each excised organ was used to calculate the distribution of the NPs in the various organs of the EAP mice.

Prostate tissue was collected and frozen to make cryosections. After sealing with an anti-fluorescence quenching tablet containing DAPI, the prostate tissue was observed by CLSM. The fluorescence intensity of the organs was measured by Living Image. Then, the fluorescence imaging results were analyzed to compare the targeting performance of the different therapeutics to the prostatitis lesion site.

### Evaluation of the therapeutic effect in vivo

One group of normal mice and 5 groups of EAP mice were prepared for modeling, with 5 mice in each group. Treatment began on the 30th day after model initiation. The normal mice were simply observed, while saline, blank NPs, free Dex, Dex/CA-Oxi-αCD NPs, or Dex/FA-CA-Oxi-αCD NPs were intravenously injected into the EAP model mice via the tail vein. The dose of Dex was 5 mg/kg (150 µL). The mice were injected with the appropriate material once every 3 days for a total of 4 administrations; the mice were weighed after each injection. At the end of the experiment, the mice were sacrificed, blood was collected and centrifuged at 3,000 rpm for 20 min at 4 °C, the supernatant was collected, and the expression levels of the inflammatory factors TNF-α, IL-1β, and IL-17 A in the blood were determined by ELISA kits.

After the mice were sacrificed, the prostate tissue was removed, a portion of which was fixed with 4% paraformaldehyde, paraffin-fixed, sliced, and stained with hematoxylin-eosin (H&E) to observe the pathological changes for determination of the treatment effect. Then, we invited two experts from the pathology department of our hospital to score the inflammation of the prostate. The other portion of prostate tissue was removed, stored at -80 °C, and tissue protein was extracted. The expression levels of the inflammatory factors TNF-α, IL-1β, and IL-17 A in the tissue were detected by WB analysis.

Additionally, the brain and L5-S1 spinal cord of each mouse were taken. The brain was fixed with 4% paraformaldehyde and sliced, and then immunohistochemical detection of the 5-HT1A receptor and SERT in the hippocampus was performed. The L5-S1 spinal cord was fixed with 4% paraformaldehyde and sliced, and then immunohistochemical detection of GFAP was performed.

### Assessment of chronic pelvic pain

We measured pelvic pain according to methods previously reported in the literature [28] (Fig. 5C). The mice were acclimated to the test environment (plexiglass box on a metal grid) for at least 30 min until they were quiet. A set of logarithmically increasing von Frey filaments (DanMic Global, LLC., San Jose, USA) was selected: 0.04 g (2.44), 0.07 g (2.83), 0.16 g (3.22), 0.4 g (3.61), 0.6 g (3.84), 1.0 g (4.08), 2.0 g (4.31), and 4.0 g (4.56) (strength and log value). The test method was as follows. First, a filament with a strength of 0.4 g was selected and stabbed vertically into the lower abdomen area near the prostate of the mouse. Next, moderate force was applied until the filament bent, and stimulation was carried out for 1 ~ 2 s each time. Positive reactions were recorded if they occurred within the test time or at the moment when the von Frey filament was removed: (1) sharp abdominal retraction; (2) immediate licking or scratching of the stimulated area of ​​the filament; (3) jumping, marked as X if there was no response, otherwise marked as O, then the adjacent smaller or larger filament was replaced to repeat the test. The interval between adjacent tests was 5 s to allow the mice to fully recover from the previous stimulation response and to avoid repeated stimulation of the same site to cause adaptation. The test’s upper and lower filament strengths were 0.04 or 4.0 g.

According to the above test method, a sequence of combinations of O or X were obtained. An O before an X appeared as the starting point, and six consecutive stimulus responses including the starting point (such as OXOXOO), were selected to calculate the 50% pelvic pain threshold. The 50% pelvic pain threshold of the mice with 4 consecutive X values or 5 O values was identified as 0.04 and 4.0 g. The 50% pelvic pain threshold of both O and X was calculated according to the following formula:

50% pelvic pain threshold (g) = (10^ (Xf + kδ))/10,000.

Here, Xf = log value of the final von Frey filament strength used; k = a constant in the table (quantitative assessment of tactile allodynia in the rat paw); and δ = average of the difference between the logarithm values ​​of the von Frey filament strength used.

### Statistical analysis

More than 3 mice were used in each in vivo experiment. All results are presented as the mean ± standard deviation. The significance of the difference between the two groups was determined by Student’s t test or Mann‒Whitney test. One-way analysis of variance (ANOVA) or the Kruskal‒Wallis test was used to analyze more than three groups. A confidence level of 0.05 was accepted as significant for all statistical tests.

## Results and discussion

### Preparation and characterization of the Dex nanoformulations

Currently, CPPS treatment still faces great challenges due to its recurrence and intractability [2]. To achieve synergistic CPPS therapy, we prepared Dex-loaded pH/ROS dual-responsive NPs using phytochemically and ROS-responsive moiety-modified α-CD as the carrier. The morphologies of these NPs were characterized by TEM (Figure S1A-C). The TEM images showed that the blank NPs, Dex/CA-Oxi-αCD NPs, and Dex/FA-CA-Oxi-αCD NPs all had spherical structures (Figure S1A-C). The physicochemical properties of the Dex-loaded NPs are listed in Table S1. The sizes of the blank NPs, Dex/CA-Oxi-αCD NPs, and Dex/FA-CA-Oxi-αCD NPs were 167.43 ± 1.68, 170.73 ± 3.23 and 179.63 ± 2.26 nm, respectively. Interestingly, compared to blank NPs, the sizes of the Dex/CA-Oxi-αCD NPs and Dex/FA-CA-Oxi-αCD NPs did not notably increase after Dex encapsulation and FA modification (Table S1). All NPs had good distribution (PDI < 0.2) with negative zeta potentials (approximately − 22 mV). The Dex content in the Dex/CA-Oxi-αCD NPs and Dex/FA-CA-Oxi-αCD NPs was 6.5% and 5.6%, respectively, suggesting that Dex can be encapsulated by the nanosystems. These NPs showed good stability in pure water and 10% FBS solution after 24 h of incubation (Figure S1G, H), indicating that these NPs can maintain their stability in vivo. These results demonstrated that we successfully fabricated pH/ROS-responsive Dex-loaded NPs with satisfactory physicochemical properties, which can be further used for in vitro or in vivo experiments. The drug release behavior from the NPs was investigated in various media. As shown in Figure S1I, compared to the PBS group, Dex release was accelerated in acidic medium (pH = 5.0) or 0.5 mM H_2_O_2_. Furthermore, drug release was clearly accelerated in acidic medium with 0.5 mM H_2_O_2_. These results implied that the fabricated NPs can release their payload in an acidic and ROS-rich microenvironment.

### Cytotoxicity of the Dex nanoformulations

Mouse macrophages, mouse prostatic epithelial cells, and human prostatic stromal cells were used to evaluate the cytotoxicity of Dex-loaded NPs using a CCK-8 kit. As shown in Figure S2A, cell viability was not obviously different when macrophages were treated with free Dex, blank NPs, Dex/CA-Oxi-αCD NPs, or Dex/FA-CA-Oxi-αCD NPs, indicating that both Dex and its nanoformulations had low toxicity to macrophages. Interestingly, the viability of prostatic epithelial cells and prostatic stromal cells notably decreased after treatment with high concentrations of Dex (Figure S2B and C). In contrast, compared to the Dex group, the viability of these cells was higher after treatment with blank NPs or the Dex nanoformulations (Figure S2B and C). These results suggest that the toxicity of Dex to these cells decreased after its encapsulation in the nanoformulations.

We also detected the intra- and extracellular H_2_O_2_ concentrations after treating cells with blank PLGA NPs (no ROS response), blank CA-Oxi-αCD NPs, Dex/CA-Oxi-αCD NPs, and Dex/FA-CA-Oxi-αCD NPs in a simulated inflammatory microenvironment. As shown in Figure S2D and E, compared to the control and PLGA NPs groups, both the intra- and extracellular H_2_O_2_ concentrations decreased significantly when the cells were treated with the blank CA-Oxi-αCD NPs and Dex nanoformulations. These results suggested that CA-Oxi-αCD NPs may eliminate ROS in prostate tissue, thereby reducing oxidative stress and alleviating the symptoms of CP.

### Cellular uptake of the Dex nanoformulations

**Fig. 2:**
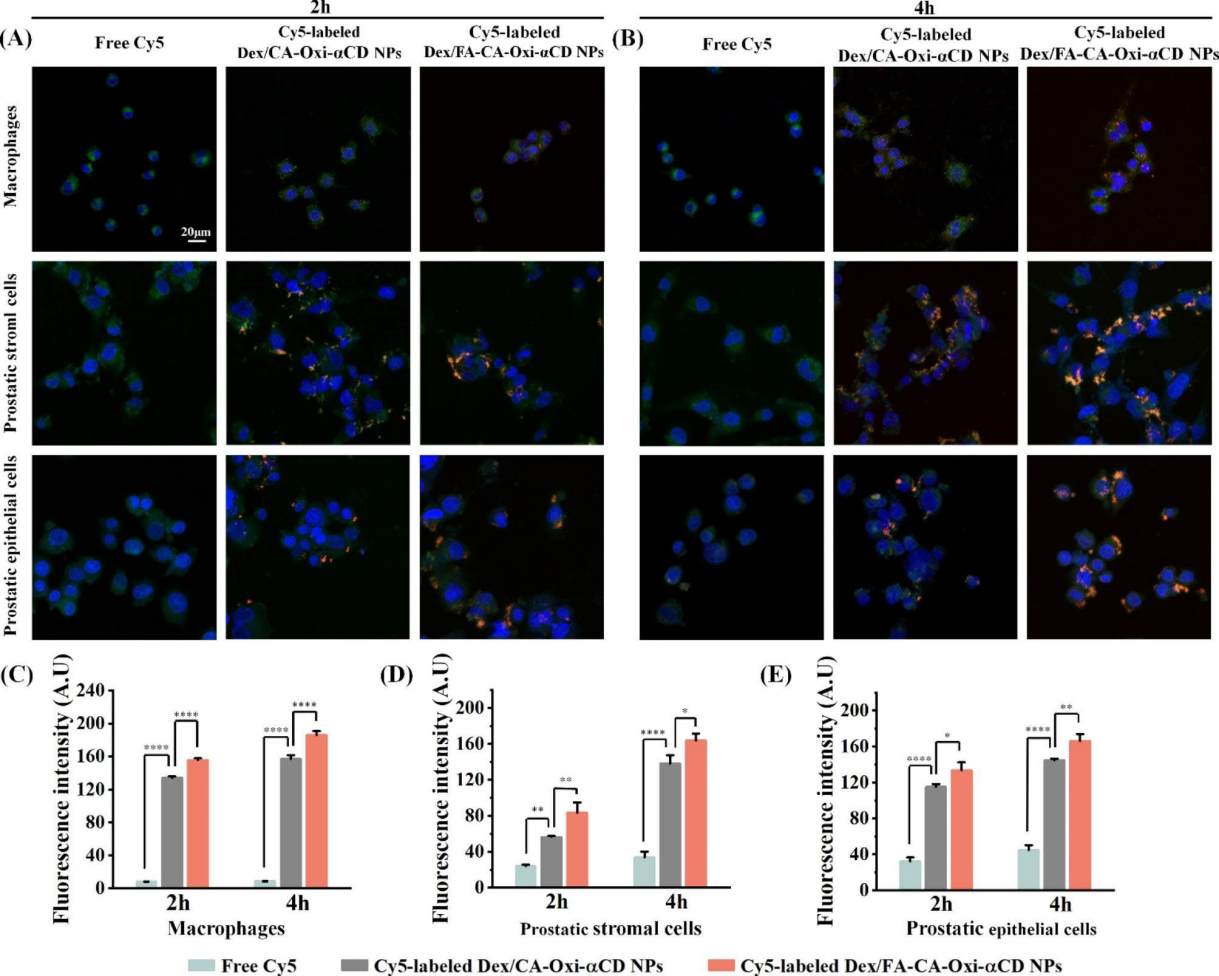
(**A, B**) CLSM images of the uptake of Cy5-labeled Dex/CA-Oxi-αCD NPs and Cy5-labeled Dex/FA-CA-Oxi-αCD NPs by LPS-stimulated macrophages, prostatic stromal cells and prostatic epithelial cells. (**C-E**) Semiquantitative analysis of the corresponding Cy5 fluorescence intensities in LPS-stimulated macrophages (**C**), prostatic stromal cells (**D**) and prostatic epithelial cells (**E**). Red indicates NPs, green indicates lysosomes and blue indicates DAPI. The scale bar represents 20 μm. ∗, Significantly different at p < 0.05; ∗∗, significantly different at p < 0.01; ∗∗∗∗, significantly different at p < 0.0001

Prostate tissue is mainly composed of prostatic epithelial cells and stromal cells, and inflammatory prostate tissue also contains infiltrated macrophages [6]. To investigate the ability of the NPs to target prostate tissue, we detected the uptake of the NPs by these types of cells. DAPI (blue) and LysoTracker (green) were used to identify the nuclei and lysosomes, respectively. As shown in Fig. 2A, only a few Cy5 fluorescence signals (red) were found in the free Cy5 group, implying that free Cy5 was hardly internalized by macrophages. In contrast, a considerable Cy5 fluorescence signal was found in the Cy5-labeled Dex/CA-Oxi-αCD NPs and Dex/FA-CA-Oxi-αCD NPs groups, indicating that these NPs can be efficiently internalized by LPS-stimulated macrophages (Fig. 2A). As expected, the Cy5-labeled Dex/FA-CA-Oxi-αCD NPs group had a higher fluorescence signal than the nontargeted group because FR overexpression was detected on the macrophages. Interestingly, after internalization, the red fluorescence signal of the NPs was highly coincident with the green fluorescence signal of LysoTracker, indicating that the internalized NPs can target lysosomes. The acidic lysosomal microenvironment can smartly control Dex release from the NPs since the nanoformulations have excellent pH responsiveness. In addition, the cellular uptake of the NPs was enhanced with prolonged incubation time (Fig. 2B). Similar results were obtained with prostatic epithelial cells and stromal cells (Fig. 2A and B). Semiquantitative analysis further demonstrated that the Dex nanoformulations can be efficiently internalized by these cells compared to free Cy5 (Fig. 2C-E). Interestingly, compared to the nontargeted NPs, the FA-modified NPs were found to have higher fluorescence intensity not only in macrophages but also in prostatic epithelial cells and stromal cells (Fig. 2C-E). Our previous work demonstrated that FR was overexpressed on inflammatory macrophages, prostatic epithelial cells and stromal cells, which exhibited strong affinity for FA [8]. Consequently, FA-modified NPs can be efficiently internalized by these cells compared to nontargeted NPs. In conclusion, our prepared Dex nanoformulations can be internalized by macrophages, prostatic epithelial cells, and stromal cells, which may cause the NPs to actively target inflammatory prostate tissue. Furthermore, internalized NPs can target lysosomes to release their payload in the acidic microenvironment.

### Cellular inflammatory factor expression after Dex nanoformulations treatment

**Fig. 3:**
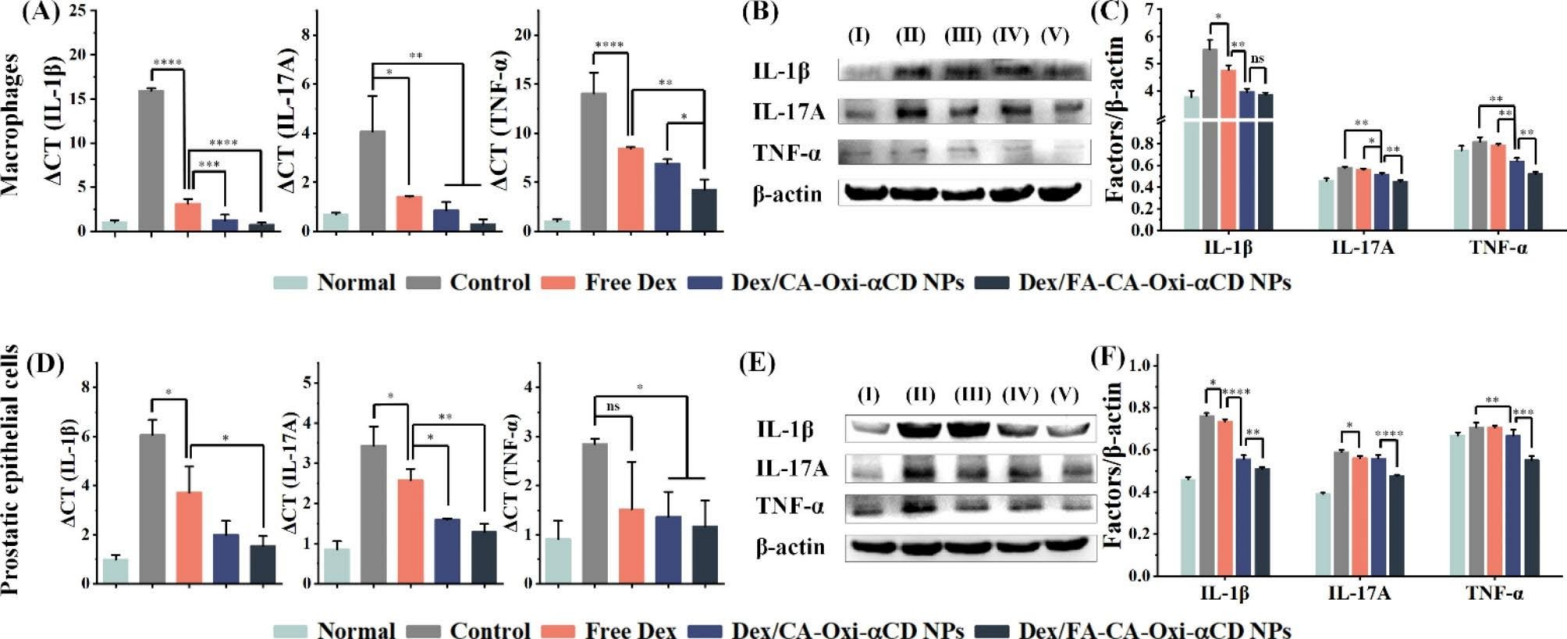
Macrophages and prostatic epithelial cells were treated with LPS and then incubated with free Dex, Dex/CA-Oxi-αCD NPs or Dex/FA-CA-Oxi-αCD NPs (Dex concentration of 5 µg/mL) for 24 h. (**A, D**) The mRNA levels of IL-1β, IL-17 A and TNF-α in macrophages and prostatic epithelial cells were measured by quantitative real-time PCR. (**B, E**) Western blot analysis of IL-1β, IL-17 A and TNF-α expression in macrophages and prostatic epithelial cells as described above. β-Actin was used as a loading control. (**C, F**) Quantitative analysis of the data presented in Fig. 3B, E. ∗, Significantly different at p < 0.05; ∗∗, significantly different at p < 0.01; ∗∗∗, significantly different at p < 0.001; ∗∗∗∗, significantly different at p < 0.0001

It has been reported that proinflammatory factors (e.g., TNF-α, IL-1β, and IL-17 A) play crucial roles in the occurrence and progression of CPPS ^[29]–[30]^. Consequently, we evaluated the ability of these NPs to regulate proinflammatory factors in macrophages and prostatic epithelial cells. The real-time PCR results showed that the levels of proinflammatory factors such as TNF-α, IL-1β, and IL-17 A were significantly increased in macrophages and prostatic epithelial cells stimulated with LPS (Fig. 2A and D). After treatment with free Dex for 24 h, the contents of inflammatory factors in these cells notably decreased compared with the control group (Fig. 2A and D), indicating that Dex can downregulate proinflammatory factor expression. Compared with the free Dex group, the expression of inflammatory factors in the cells treated with the Dex-loaded NPs was further downregulated, suggesting that delivery via NPs enhanced the anti-inflammatory effect of Dex. The anti-inflammatory activities of the NPs in cells were further investigated by WB experiments. As shown in Fig. 3B and E, strong black bands were found in the control group compared to the normal group, indicating that proinflammatory factors were overexpressed after LPS stimulation. This band was attenuated after Dex treatment, suggesting that Dex can decrease proinflammatory mediator expression in these cells. Interestingly, the bands were further attenuated by treatment with the Dex nanoformulations compared to free Dex group, indicating that the nanoformulations can enhance the anti-inflammatory activity of Dex (Fig. 3B and E). Semiquantitative analysis of the bands verified that Dex/FA-CA-Oxi-αCD NPs displayed better anti-inflammatory effects than free Dex or its nontargeted nanoformulations (Fig. 3C and F). As mentioned above, FA-modified Dex/CA-Oxi-αCD NPs can be effectively internalized by macrophages and prostatic epithelial cells, thereby releasing more therapeutics to reduce inflammatory factor expression. In addition, we detected apoptosis of LPS-stimulated prostatic cells and stromal cells incubated with Dex and its nanoformulations. As shown in Figure S3, FA-modified Dex/CA-Oxi-αCD NPs clearly induced inflammatory cell apoptosis compared to Dex or Dex/CA-Oxi-αCD NPs, which may contribute to the enhanced anti-inflammatory activity of Dex/FA-CA-Oxi-αCD NPs.

### In vivo distribution of the Dex nanoformulations

**Fig. 4:**
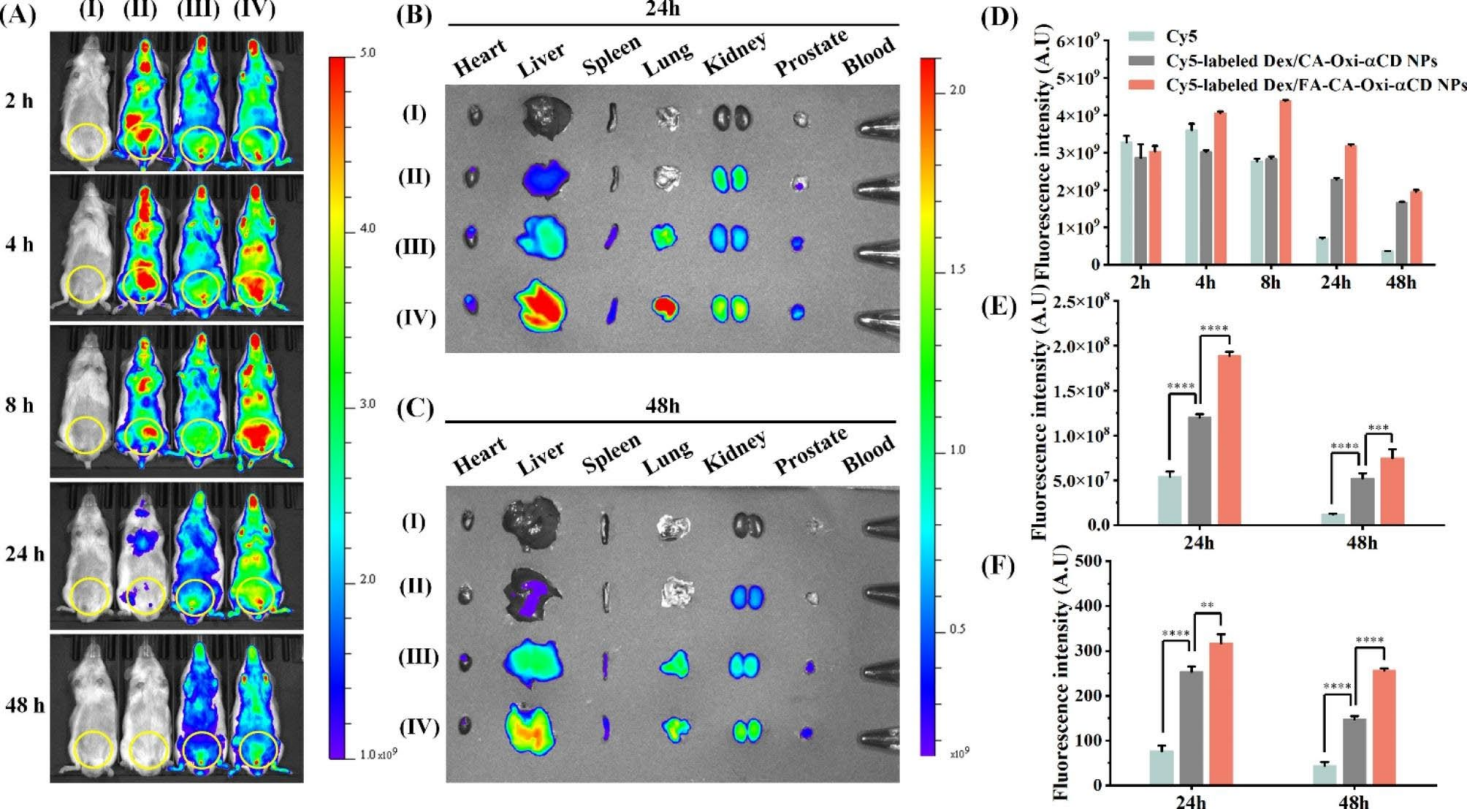
In vivo distribution of Cy5-labeled Dex/CA-Oxi-αCD NPs and Cy5-labeled Dex/FA-CA-Oxi-αCD NPs in EAP model mice. (**A**) In vivo fluorescence images of saline (I), free Cy5 (II), Cy5-labeled Dex/CA-Oxi-αCD NPs (III) and Cy5-labeled Dex/FA-CA-Oxi-αCD NPs (IV) in EAP model mice at different time points after intravenous injection (the region in the yellow circle indicates the lower urinary tract and part of the reproductive system). (**B-C**) Ex vivo fluorescence images of the excised major organs and prostates at 24 h (**B**) and 48 h (**C**) postinjection: heart, liver, spleen, lung, kidney, prostate and blood. (**D**) ROI analysis of the fluorescence intensity in the lower urinary tract and part of the reproductive system was performed at different time points after intravenous injection. (**E**) ROI analysis of the fluorescence intensity of the excised prostates at 24 and 48 h. (**F**) Semiquantitative analysis of the CLSM images of prostate tissues treated with Cy5 or Cy5-labeled NPs for 24 and 48 h. ∗∗, significantly different at p < 0.01; ∗∗∗, significantly different at p < 0.001; ∗∗∗∗, significantly different at p < 0.0001

Our previous work demonstrated that FA-modified NPs can penetrate the prostatic epithelium and effectively deliver antibiotics to the glandular lumen in bacteria-infected prostate tissues [8]. Herein, to investigate whether FA-modified pH/ROS dual-responsive NPs can target inflammatory prostate tissue, we observed the in vivo distribution of NPs in EAP mice using a live imaging system. The field of observation was near the lower urethral area, including the prostate. As shown in Fig. 4A, when EAP mice received free Cy5, the strongest fluorescence intensity appeared at 4 h after administration and decreased significantly at 24 h after administration, indicating that free Cy5 was rapidly excreted from the body after injection (Fig. 4A). However, considerable fluorescence intensities continued to be observed in the lower urinary tract and part of the reproductive system at 24 and 48 h after EAP mice were injected with Cy5-labeled NPs. These results suggested that Cy5-labeled NPs have a longer in vivo circulation time than free Cy5 (Fig. 4A). Semiquantitative analysis also showed that the Cy5-labeled NPs exhibited higher fluorescence intensity than free Cy5 in EAP mice at 24 and 48 h after administration (Fig. 4D). To further investigate the in vivo distribution of the NPs, mice were sacrificed at 24 and 48 h after administration, and their blood, internal organs, and prostate tissues were collected and observed by an imaging system. As shown in Fig. 4B and C, weak fluorescence was observed in the prostate tissue when EAP mice received free Cy5. In contrast, considerable fluorescence intensity was found in the prostate tissues of mice administered Cy5-labeled NPs (Fig. 4B and C), indicating that the Dex nanoformulations can effectively accumulate in prostate tissues. As expected, the FA-modified NPs group exhibited stronger fluorescence intensity in the prostate tissue than the nontargeted NPs group (Fig. 4B and C). Semiquantitative analysis also demonstrated that the Cy5-labeled Dex/FA-CA-Oxi-αCD NPs produced stronger fluorescence intensity in prostate tissue than free Cy5 or the Cy5-labeled Dex/CA-Oxi-αCD NPs (Fig. 4E).

To further investigate the NPs targeting ability, the excised prostate tissue was frozen and stained with DAPI and observed by CLSM. As shown in Figure S4, the fluorescence intensity in the prostate tissue from the free Cy5 group was very weak. However, a strong fluorescence signal was observed in the intraluminal space of the prostate tissue from the mice injected with Cy5-labeled NPs (Figure S4), indicating that Cy5-labeled NPs can target the glandular lumen of prostate tissue. Semiquantitative analysis also demonstrated that the Cy5-labeled Dex/FA-CA-Oxi-αCD NPs group had higher fluorescence intensity in the glandular lumen of prostate tissue than the Cy5-labeled Dex/CA-Oxi-αCD NPs group (Fig. 4F). The above results suggested that Dex nanoformulations, especially the FA-modified NPs, can effectively accumulate in the glandular lumen of inflamed prostate tissue.

### In vivo therapeutic efficacy of the Dex nanoformulations in EAP mice

According to the theory of immune pathogenesis, a rodent EAP model was established by invoking an autoimmune response to many antigens [27]. EAP mice are characterized by prostate-specific cellular and humoral immune responses that are accompanied by typical histological infiltrative lesions, similar to those in human patients [31]. Additionally, rodent models are associated with signs of chronic pelvic pain [32]. Because the symptoms, pathology, and immunological characteristics of the EAP model are highly consistent with those of human CP/CPPS, EAP mice are ideal models for the study of CP [33]. Therefore, in the current study, we selected NOD mice to establish an EAP model to evaluate the efficacy of the Dex nanoformulations on CPPS.

**Fig. 5:**
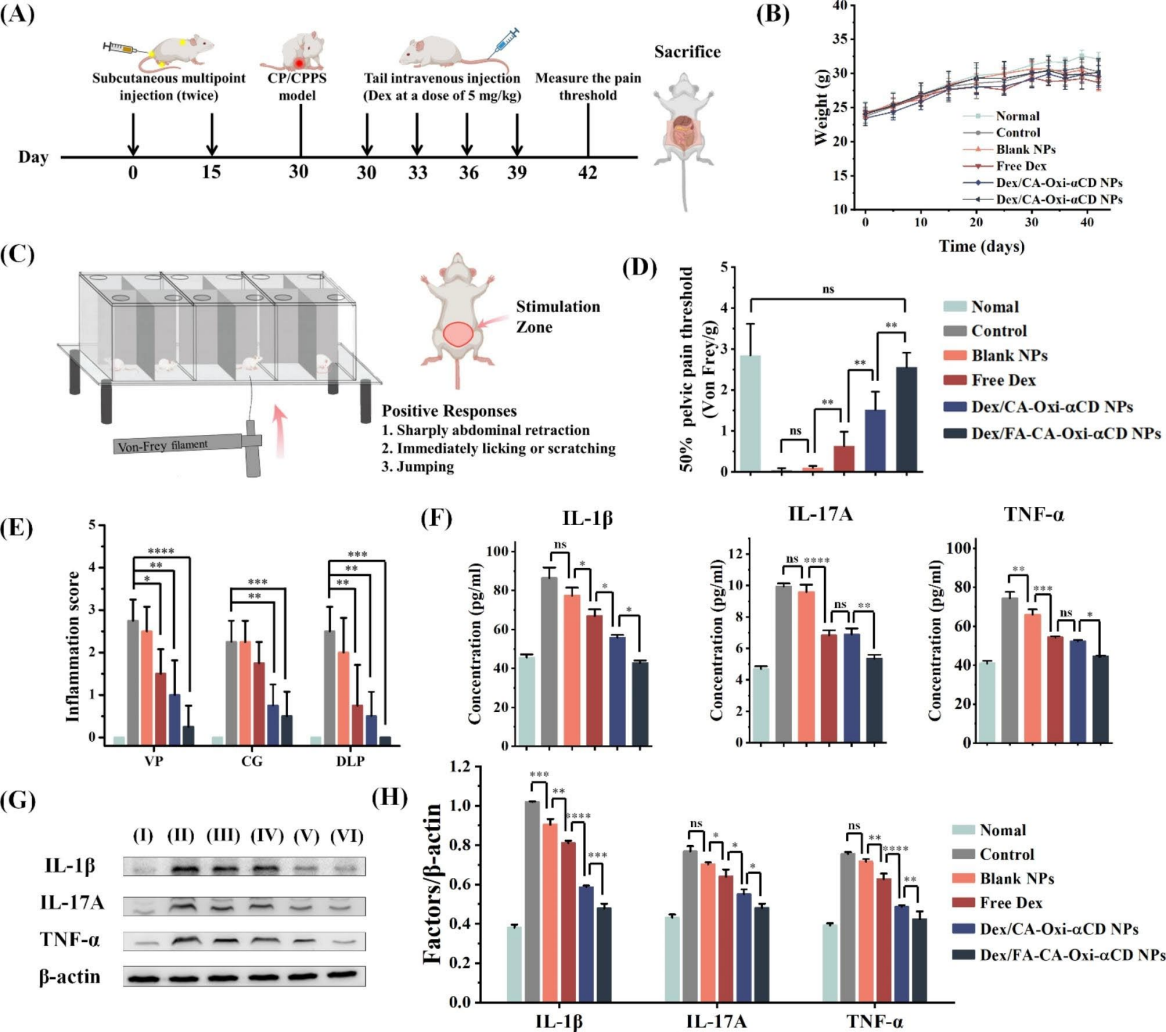
(**A**) Schematic diagram of the EAP mouse modeling and treatment processes. (**B**) Body weights of the mice. (**C**) Graphical depiction of von Frey filament testing to determine pelvic pain. (**D**) The pelvic pain of the mice was measured with von Frey filaments on the 3rd day after the last injection. (**E**) Inflammation scores of the prostate tissues after treatment. The mouse prostate consists of the ventral prostate (VP), dorsal and lateral prostate (DLP), and anterior or coagulating gland (CG) lobes. (**F**) The IL-1β, IL-17 A and TNF-α levels in the serum of mice after treatment determined with ELISA kits. (**G**) Western blot analysis of IL-1β, IL-17 A and TNF-α expression in the prostate tissues from EAP mice that had been subjected to various treatments. β-Actin was used as a loading control. (**H**) Quantitative analysis of the data presented in Fig. 5G. ∗, Significantly different at p < 0.05; ∗∗, significantly different at p < 0.01; ∗∗∗, significantly different at p < 0.001; ∗∗∗∗, significantly different at p < 0.001

The model building strategy and dosages of therapeutics are listed in Fig. 5A. During treatment, there were no significant differences in body weight among the treatment groups (Fig. 5B). After finishing treatment, we used von Frey filaments to measure the 50% pelvic pain threshold of each mouse (Fig. 5C). Compared with the normal group, the pain threshold of the mice in the control group was significantly decreased, indicating that these EAP mice had clear pelvic pain symptoms. The pain threshold of mice was elevated by blank NPs treatment compared with that of the control group mice, but there was no significant difference between the two groups (Fig. 5D). The pelvic pain threshold in the free Dex group of mice was slightly higher than that in the blank NPs or control group, indicating that Dex can alleviate pelvic pain in mice (Fig. 5D). Interestingly, the pelvic pain threshold of the mice in the Dex-loaded NP group was significantly increased compared to that of the mice in the free Dex group, suggesting that the Dex nanoformulations can notably relieve CPPS symptoms (Fig. 5D). Dex/FA-CA-Oxi-αCD NPs distinctly elevated the pain threshold of mice compared to the other treatments, since FA-modified NPs can effectively accumulate in inflammatory prostate tissue to eliminate ROS or release anti-inflammatory molecules to alleviate CPPS symptoms (Fig. 5D).

**Fig. 6:**
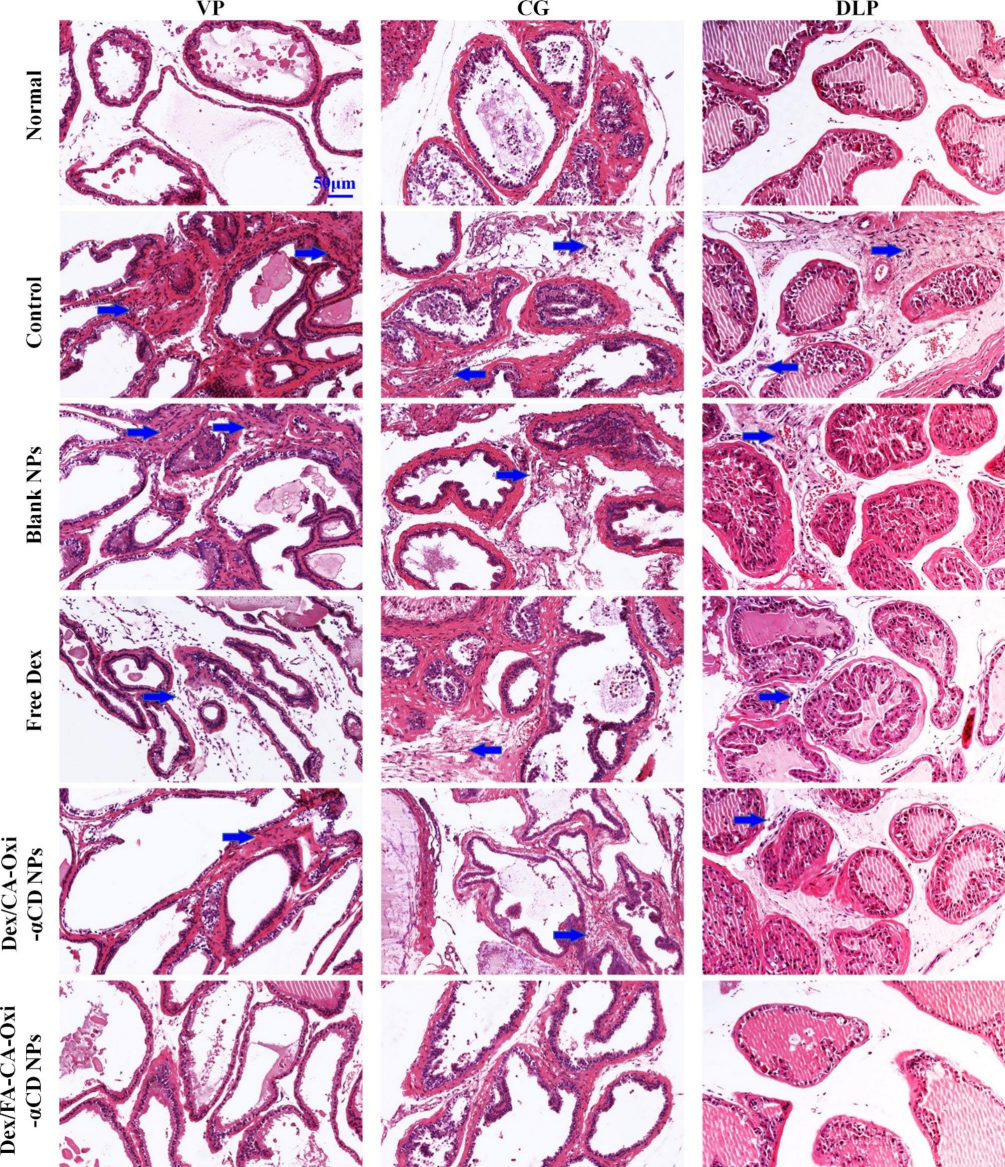
H&E-stained sections of prostate tissues (ventral prostate (VP), dorsal and lateral prostate (DLP), coagulating gland (CG)) from normal and EAP mice treated with saline, blank NPs, free Dex, Dex/CA-Oxi-αCD NPs and Dex/FA-CA-Oxi-αCD NPs. The blue arrow indicates inflammatory cell infiltration. Scale bar represents 50 μm

Histopathological analysis of prostate tissues provided visual evidence to evaluate the inflammatory response. Consequently, after treatment, the prostate tissues of EAP mice were collected and observed by H&E staining. As shown in Fig. 6, the serious inflammatory cell infiltration and partial tissue damage in the prostatic interstitium, glands, and surrounding glands in the VP lobes of the model mice indicated that the EAP model was successfully established, as these signs were absent in normal mice. The inflammatory response in the blank NPs group was not significantly relieved compared with that in the control group (Fig. 6), while the infiltration of inflammatory cells in the free Dex group was partially decreased because Dex is a classical steroidal anti-inflammatory drug. Importantly, the inflammation of prostate tissues in the Dex/CA-Oxi-αCD NPs and Dex/FA-CA-Oxi-αCD NPs groups was significantly relieved, and the number of inflammatory cells that had infiltrated the stroma was significantly reduced (Fig. 6). The prostatic inflammation scores of the VP, CG, and DLP lobes based on H&E staining also demonstrated that the FA-modified Dex/CA-Oxi-αCD NPs had the best anti-inflammatory effect compared to other therapeutics (Fig. 5E). In conclusion, in vivo animal experiments demonstrated that FA-modified Dex/CA-Oxi-αCD NPs can distinctly alleviate the symptoms of CPPS.

Moreover, we initially evaluated the biological safety of the nanoformulations in vivo. The routine blood data showed that there were no significant differences in red blood cell count, platelet count, or hemoglobin level between the normal group and treatment groups (Figure S5A-C). The white blood cell (WBC) counts in the control group were higher than those in the normal group (Figure S5D) since the inflammatory response may elevate the WBC content. However, the WBC content notably decreased in the treatment groups (Figure S5D) because the inflammatory response was alleviated with therapeutic treatment. The levels of ALT, AST, URE, and CRE, which are related to liver and kidney function, were not significantly different between the normal and treatment groups (Figure S5E and F). These results showed that the administration of Dex nanoformulations in vivo has no significant hematological adverse effects. Then, we observed the pathological changes in the hearts, livers, spleens, lungs, and kidneys of the mice by H&E staining. Compared to the normal group, there was no obvious tissue damage or inflammatory cell infiltration in the free Dex and Dex nanoformulations groups, suggesting that these nanoformulations did not have obvious systemic toxicity. In summary, the Dex nanoformulations had considerable safety in mice at a dose of 5 mg/kg.

### Anti-inflammatory mechanism of the Dex nanoformulations in EAP mice

In vivo experiments verified that the Dex nanoformulations can significantly relieve CPPS in EAP mice, which inspired us to further investigate the anti-inflammatory mechanism of these NPs in EAP mice. Proinflammatory cytokines, including TNF-α and IL-1β, play a crucial role in the pathogenesis of CP, and their expression is closely related to the inflammatory response and prostate tissue destruction [34]. In addition, IL-17 plays an important part in the pathogenesis of EAP and may indirectly cause pelvic pain associated with CP [29]. Therefore, the expression of these inflammatory cytokines in the plasma and prostate tissue of EAP mice was detected after treatment. The ELISA results showed that the concentrations of TNF-α, IL-1β, and IL-17 A in the serum of the model group were significantly higher than those in the normal group (Fig. 5F), which further demonstrated that the EAP model was successfully established. Blank NPs treatment decreased TNF-α expression (Fig. 5F) because it eliminated ROS and released the phytochemical CA, which alleviated the inflammatory response. After free Dex and Dex-loaded NPs treatment, the expression of IL-1β, IL-17 A, and TNF-α in the serum of mice clearly decreased (Fig. 5F), suggesting that Dex or its nanoformulations can downregulate inflammatory cytokine expression to relieve the inflammatory response. Dex/FA-CA-Oxi-αCD NPs significantly inhibited the expression of these inflammatory factors in serum compared to the other therapeutics (Fig. 5F) due to their enhanced ability to target inflammatory prostate tissues. These results demonstrated that the Dex nanoformulations, especially the FA-modified Dex nanoformulations, can downregulate the expression of proinflammatory factors such as IL-1β, IL-17 A, and TNF-α in serum to alleviate the symptoms of CPPS.

The expression of TNF-α, IL-1β, and IL-17 A in the prostate tissue of EAP mice was also detected by WB analysis (Fig. 5G and H). Compared with normal mice, the prostate tissue of EAP mice displayed significantly enhanced bands of TNF-α, IL-1β, and IL-17 A (Fig. 5G and H). When EAP mice were treated with blank NPs, the expression of IL-1β in prostate tissues was downregulated compared to that in the model group. These results further verified that the synthesized materials have certain anti-inflammatory activity. In accordance with the serum results, Dex notably decreased the proinflammatory factor expression level in the prostate tissues of EAP mice (Fig. 5G and H). Interestingly, both the nontargeted and targeted Dex nanoformulations significantly decreased the expression of TNF-α, IL-1β, and IL-17 A in the prostate tissues of EAP mice compared to free Dex (Fig. 5G and H), demonstrating that the Dex nanoformulations displayed better anti-inflammatory activity than Dex for CPPS treatment. Importantly, the expression of these proinflammatory factors in the prostate tissues of EAP mice treated with Dex/FA-CA-Oxi-αCD NPs was almost equivalent to that in normal mice (Fig. 5G and H), suggesting that the CPPS in mice was cured with Dex/FA-CA-Oxi-αCD NP treatment. In conclusion, these Dex nanoformulations can effectively reduce the expression of proinflammatory factors such as TNF-α, IL-1β, and IL-17 A in EAP mice to alleviate the symptoms of prostatitis.

**Fig. 7:**
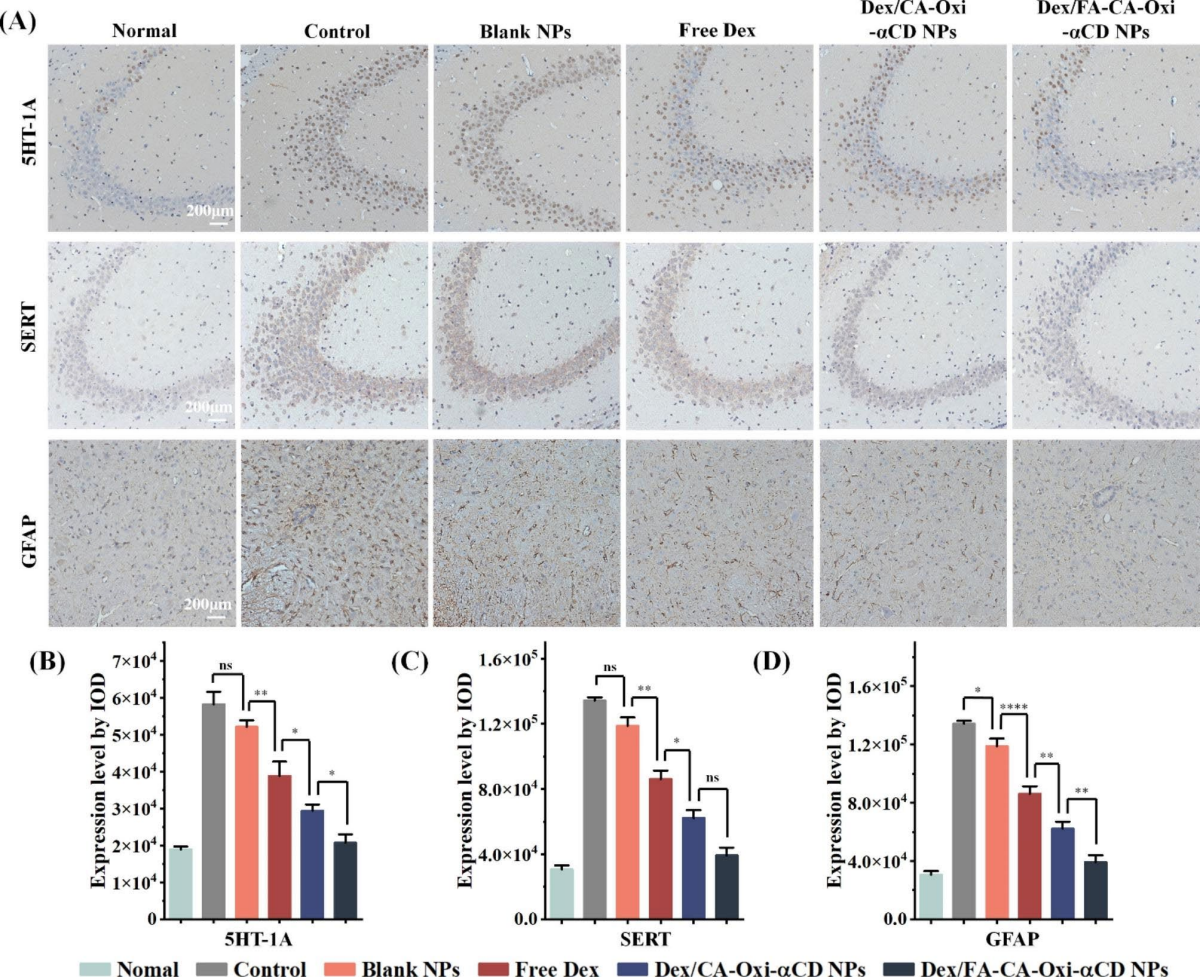
(**A**) Immunohistochemistry of the 5-HT1A receptor and SERT in the mouse hippocampus and GFAP in the L5-S1 segment of the spinal cord. (B-D) Semiquantitative absorbance values of the 5-HT1A receptor (**B**), SERT (**C**) and GFAP (**D**). Scale bar represents 200 μm. ∗, Significantly different at p < 0.05; ∗∗, significantly different at p < 0.01; ∗∗∗∗, significantly different at p < 0.001

It has been reported that patients with depression have 5-HT1A receptor and SERT overexpression, and CPPS may cause patient depression ^[35]–[36]^. To investigate whether curing CPPS with these Dex nanoformulations should alleviate depression in patients, we detected 5-HT1A receptor and SERT expression in the brain tissues of EAP mice using immunohistochemical staining. As shown in Fig. 7A-C, the 5-HT1A receptor and SERT contents in the brain tissues of EAP mice were notably elevated compared to those in normal mice, indicating that EAP mice may have depression. Interestingly, the expression of the 5-HT1A receptor and SERT was significantly downregulated when the EAP mice were treated with Dex or its nanoformulations compared to that in the model group mice (Fig. 7A-C), suggesting that alleviating CPPS may relieve depression in mice. Importantly, after Dex/FA-CA-Oxi-αCD NPs treatment, the expression of the 5-HT1A receptor and SERT in the brain tissues of EAP mice was similar to that in normal mice. As mentioned above, Dex/FA-CA-Oxi-αCD NPs can cure the symptoms of CPPS in mice, which may cause depression in mice to disappear.

In addition, the expression of GFAP in the L5-S1 spinal cord is related to pain. Consequently, the GFAP content in the spinal cord of EAP mice was evaluated using immunohistochemical staining. As shown in Fig. 7A and D, the levels of GFAP in the spinal cord of the model group were significantly higher than those in the normal group, indicating that the model mice had pelvic pain. Dex or its nanoformulations clearly downregulated the expression of GFAP, suggesting that the pain of EAP mice was alleviated after treatment with Dex or its nanoformulations. Interestingly, the expression of GFAP in the Dex/FA-CA-Oxi-αCD NPs group was similar to that in the normal group (Fig. 7A and D), indicating that the pain in EAP mice had almost disappeared. These results are in accordance with those from the pain threshold experiments.

## Conclusion

The discomfort caused by CP/CPPS greatly affects patients’ quality of life, and a strategy for the effective management of CPPS is still lacking. In addition, oxidative stress can further aggravate prostatitis symptoms. Steroid anti-inflammatory drugs have shown good efficacy against CPPS; however, their adverse effects should not be ignored. To develop a novel therapeutic for the management of CPPS, we fabricated Dex nanoformulations using pH/ROS dual-responsive materials.

In vitro experiments demonstrated that the Dex nanoformulations can release their payload in acidic and/or ROS-rich microenvironments. In vitro cellular assays verified that the Dex nanoformulations, especially the FA-modified Dex nanoformulations, can be efficiently internalized by LPS-stimulated macrophages, prostatic epithelial cells, and prostatic stromal cells. The internalized nanoformulations can decrease the expression levels of proinflammatory factors (i.e., TNF-α, IL-1β, and IL-17 A) in these cells because they can eliminate ROS in inflammatory prostate tissues and release the anti-inflammatory drug Dex, and phytochemical CA, which synergistically alleviates inflammation. Flow cytometry also demonstrated that the Dex nanoformulations can increase the apoptosis rate of inflammatory cells, which may enhance the anti-inflammatory effect of Dex.

In vivo imaging showed that the FA-modified Dex nanoformulations can effectively penetrate the prostatic epithelium and accumulate in the glandular lumen. In vivo experiments also demonstrated that the Dex nanoformulations can effectively reduce prostate tissue inflammation to alleviate pelvic pain. Compared to free Dex, the FA-modified Dex nanoformulations distinctly decreased the expression of proinflammatory cytokines (e.g., TNF-α, IL-1β, and IL-17 A) in the plasma and prostate tissues of mice to relieve the inflammatory response. The above results indicated that Dex-loaded NPs could target inflamed prostate tissue and effectively reduce the expression of inflammatory factors to alleviate the pathological changes in prostatitis and relieve the symptoms of pelvic pain.

The serotonin system plays an important role in depression and male sexual dysfunction [37]. Previous studies have demonstrated that the level of the 5-HT1A receptor is associated with the pathogenesis of depression [35]. SERT promotes serotonin uptake and decreases serotonin levels [36]. The expression levels of the 5-HT1A receptor and SERT increased, which may be related to depression and sexual dysfunction [38]. The expression of the serotonin system also changed in the EAP model. Immunohistochemistry showed that the levels of the 5-HT1A receptor and SERT in the hippocampus of the EAP mice increased significantly. After treatment with the Dex nanoformulations, their expression in EAP mice was similar to that in normal mice. Moreover, we also found that the expression of GFAP in the L5-S1 spinal cord of EAP mice was significantly upregulated, while it was clearly downregulated after treatment with the Dex nanoformulations. These results suggested that CP may lead to depression and sexual dysfunction in mice through the serotonin system and upregulate GFAP in the spinal cord, leading to pelvic pain. Thus, these Dex nanoformulations may inhibit the inflammatory response in the prostate to relieve depression and pelvic pain in mice. Although we did not investigate the behavior and mechanism of these actions further, our results provide a practicable strategy to alleviate CPPS in mice, which may also relieve depression in mice.

## Electronic supplementary material

Below is the link to the electronic supplementary material.

Supplementary Material 1
