# Supplementary material for: Glucocorticoid nanoformulations relieve chronic pelvic pain syndrome and may alleviate depression in mice
Source: J Nanobiotechnology. 2023 Jun 21;21:198. doi: 10.1186/s12951-023-01893-4 (PMC10283268; doi:10.1186/s12951-023-01893-4)
Supplement: Supplementary file 1 — Supplementary Material 1 [file 12951_2023_1893_MOESM1_ESM.docx]

**Supporting Information**

**1. Materials synthesis**

**1.1 Degradation and anti-inflammatory mechanism of pH/ROS dual-responsive materials**

Scheme S1. Degradation and anti-inflammatory mechanism of CA-Oxi-αCD under acidic or ROS-rich conditions.

**1.2 Reagents**

All chemical reagents and anhydrous solvents were obtained from commercial sources and used directly without further purification. α-Cyclodextrin (α-CD) and cinnamaldehyde (CA) were purchased from Aladdin Chemical Co., Ltd. (Shanghai, China). Pyridinium p-toluenesulfonate (PPTS) was provided by J&K Scientific Co., Ltd. (Beijing, China). 1-(3-Dimethylaminopropyl)-3-ethylcarbodiimide hydrochloride (EDC·HCl), 4-(hydroxymethyl) phenylboronic acid pinacol ester (HPAP), 4-dimethylaminopyridine (DMAP), 1,1-carbonyldiimidazole (CDI) and anhydrous dimethyl sulfoxide (DMSO) were obtained from Sigma‒Aldrich. (St. Louis, USA). Trimethoxymethane and other organic solvents were received from Chengdu Kelong Chemicals Co., Ltd. (Chengdu, China).

**1.3 Synthesis of CA-modified α-CD (CA-αCD)**

Scheme S2. Synthesis of CA-modified α-CD

CA-modified α-CD was synthesized by reacting cinnamon acetal (CAA) with α-CD (Scheme 1). In brief, 5.0 g of CA, 19.4 g of trimethoxymethane, and 2.0 g of PPTS were dissolved in 50 mL of methanol and refluxed for 3 h at 62 °C. After quenching the reaction with a saturated NaHCO_3_ solution, the reaction mixture was extracted with ethyl acetate, and the organic layer was collected and dried over MgSO_4_. After removing the solvent, a yellow liquid product (7.41 g) was harvested and used for the next step.

α-CD (1.6 g) and 3.52 g of CAA were dissolved in 14 mL of anhydrous DMSO, and 0.25 g of PPTS was added to the solution. After 72 h of reaction at 60 °C under an argon atmosphere, the mixture was precipitated with cold acetone, and a white powder (1.73 g) was obtained after drying in a vacuum oven.

**1.4 Synthesis of the pH/ROS dual-responsive materials (CA-HPAP-αCD)**

Scheme S3. Synthesis of the CA-HPAP-αCD materials

HPAP (2.77 g) was dissolved in 18 mL of dry dichloromethane (DCM), and then 3.83 g of CDI was added. After the reaction was complete, 20 mL of DCM was added, and the mixture was washed with 30 mL of deionized water three times. Finally, 3.43 g of CDI-activated HPAP was obtained when the solvent was removed.

CA-αCD (1.0 g) was dissolved in 30 mL of anhydrous DMSO, and then 2.22 g of DMAP was added. CDI-activated HPAP (1.18 g) was then added to the above solution. After 72 h of reaction, the crude product was obtained by precipitation with 40 mL of water and centrifugation. The crude product was further purified by dialysis for 2 days in pure water (MWCO: 1000 Da).

**2. Table**

Table S1. Size, PDI, zeta potential, and drug loading of the blank NPs, Dex/CA-Oxi-αCD NPs, and Dex/FA-CA-Oxi-αCD NPs. Data represent the mean ± SD (n = 3).

| Nanoformulations | Size (nm) | PDI | Zeta potential (mV) | Drug loading (%) |
| --- | --- | --- | --- | --- |
| Blank NPs | 167.4 ± 1.7 | 0.18 | -22.2 ± 0.9 |  |
| Dex/CA-Oxi-αCD NPs | 170.7 ± 3.2 | 0.19 | -22.1 ± 0.3 | 6.5 |
| Dex/FA-CA-Oxi-αCD NPs | 179.6 ± 2.2 | 0.18 | -22.7 ± 1.1 | 5.6 |

**3. Figures**


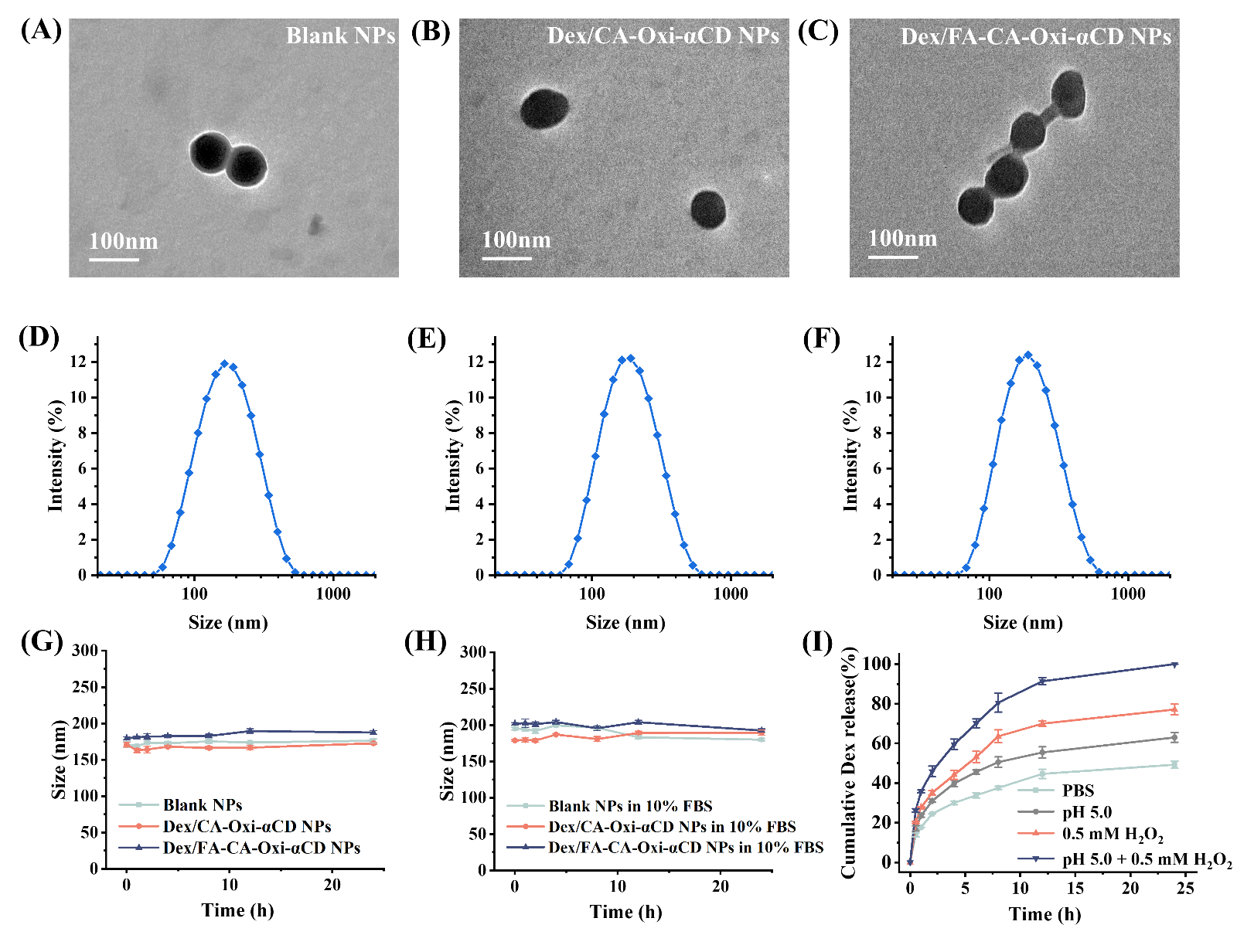


Figure S1. (A-F) TEM images and size distributions of the blank NPs (A, D), Dex/CA-Oxi-αCD NPs (B, E), and Dex/FA-Oxi-αCD NPs (C, F). The morphology of the NPs was observed by TEM (A, B, C). The size distributions of the NPs were measured by DLS (D, E, F). (G, H) Stability of the blank NPs, Dex/CA-Oxi-αCD NPs, and Dex/FA-CA-Oxi-αCD NPs in pure water or 10 % FBS solution. (I) Drug release from the Dex/CA-Oxi-αCD NPs in PBS, pH 5.0/PBS, 0.5 mM H_2_O_2_/PBS, or pH 5.0+0.5 mM H_2_O_2_/PBS.


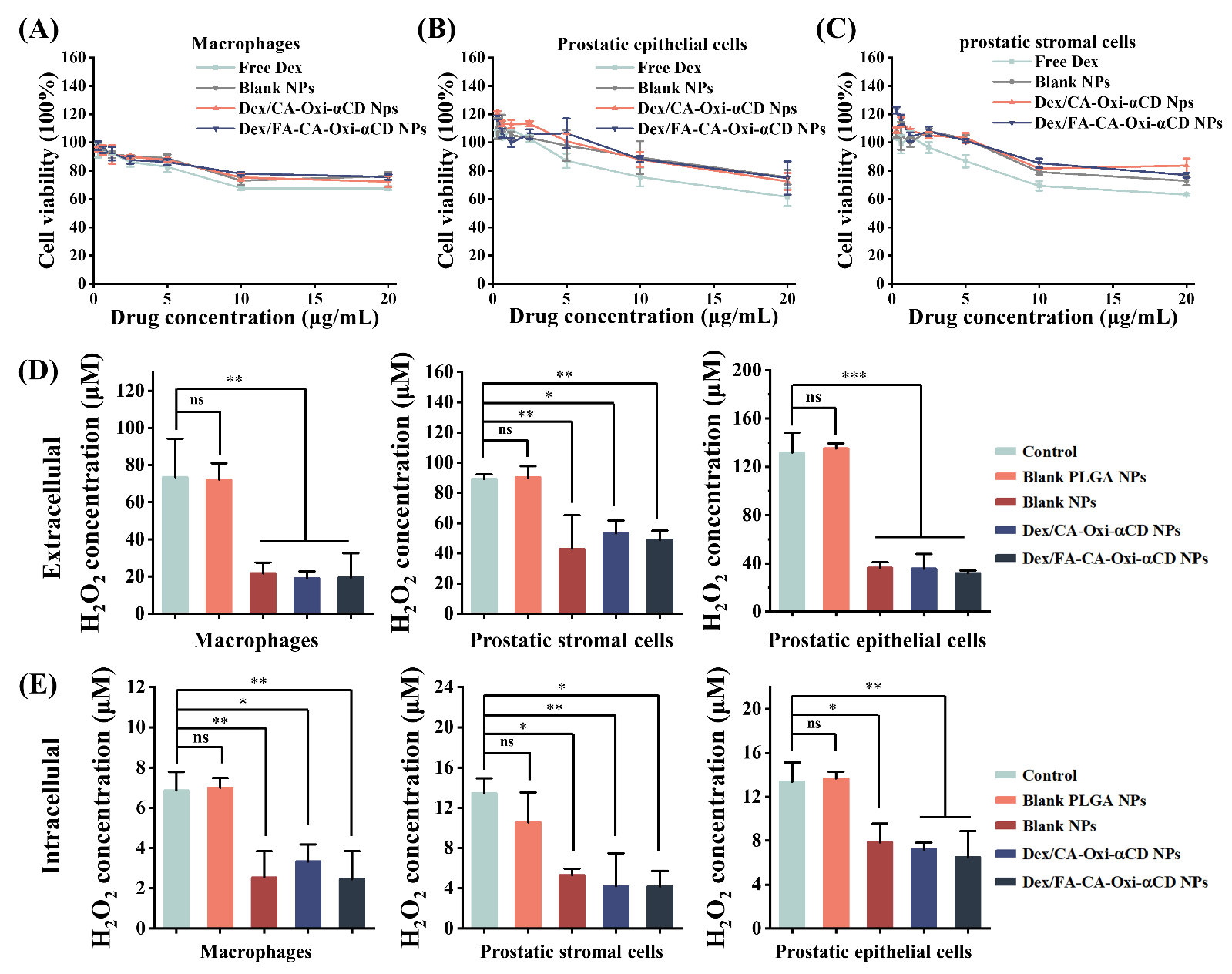


Figure S2. (A-C) Toxicity of Dex/CA-Oxi-αCD NPs and Dex/FA-CA-Oxi-αCD NPs to macrophages (A), prostatic stromal cells (B), and prostatic epithelial cells (C) determined by CCK-8 assay. Inflammatory macrophage, prostatic stromal cell, and prostatic epithelial cell extracellular (D) and intracellular (E) H_2_O_2_ concentrations measured after nanoformulations treatment. ∗, Significantly different at p < 0.05; ∗∗, significantly different at p < 0.01; ∗∗∗, significantly different at p < 0.001.


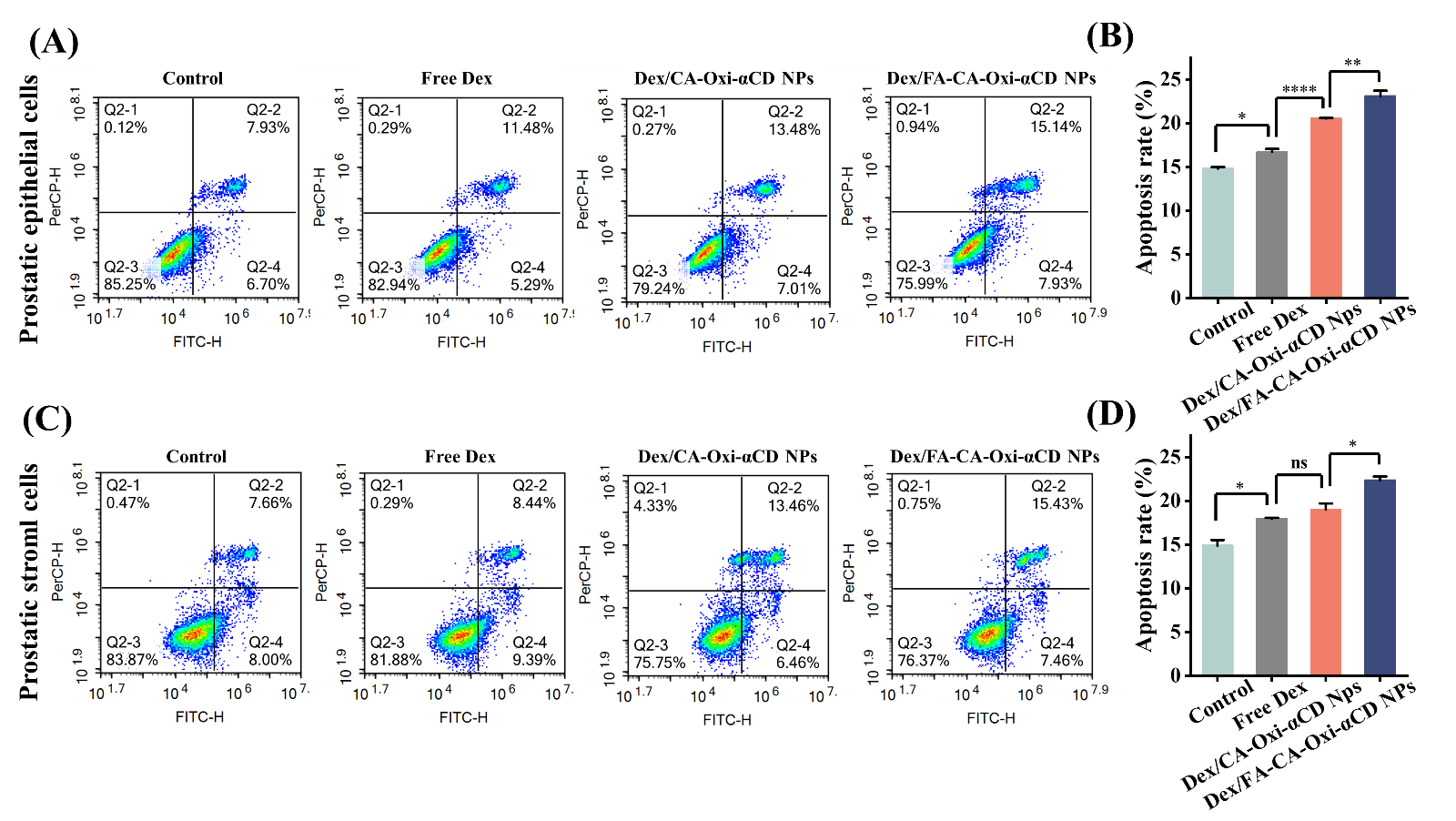


Figure S3. The apoptosis rates of inflammatory mouse prostatic epithelial cells (A) and human prostatic stromal cells (C) were detected by flow cytometry with Dex or its nanoformulations treatment. Analysis of the overall apoptosis rates of mouse prostate epithelial cells (B) and human prostatic stromal cells (D). ∗, Significantly different at p < 0.05; ∗∗, significantly different at p < 0.01; ∗∗∗∗, significantly different at p < 0.001.


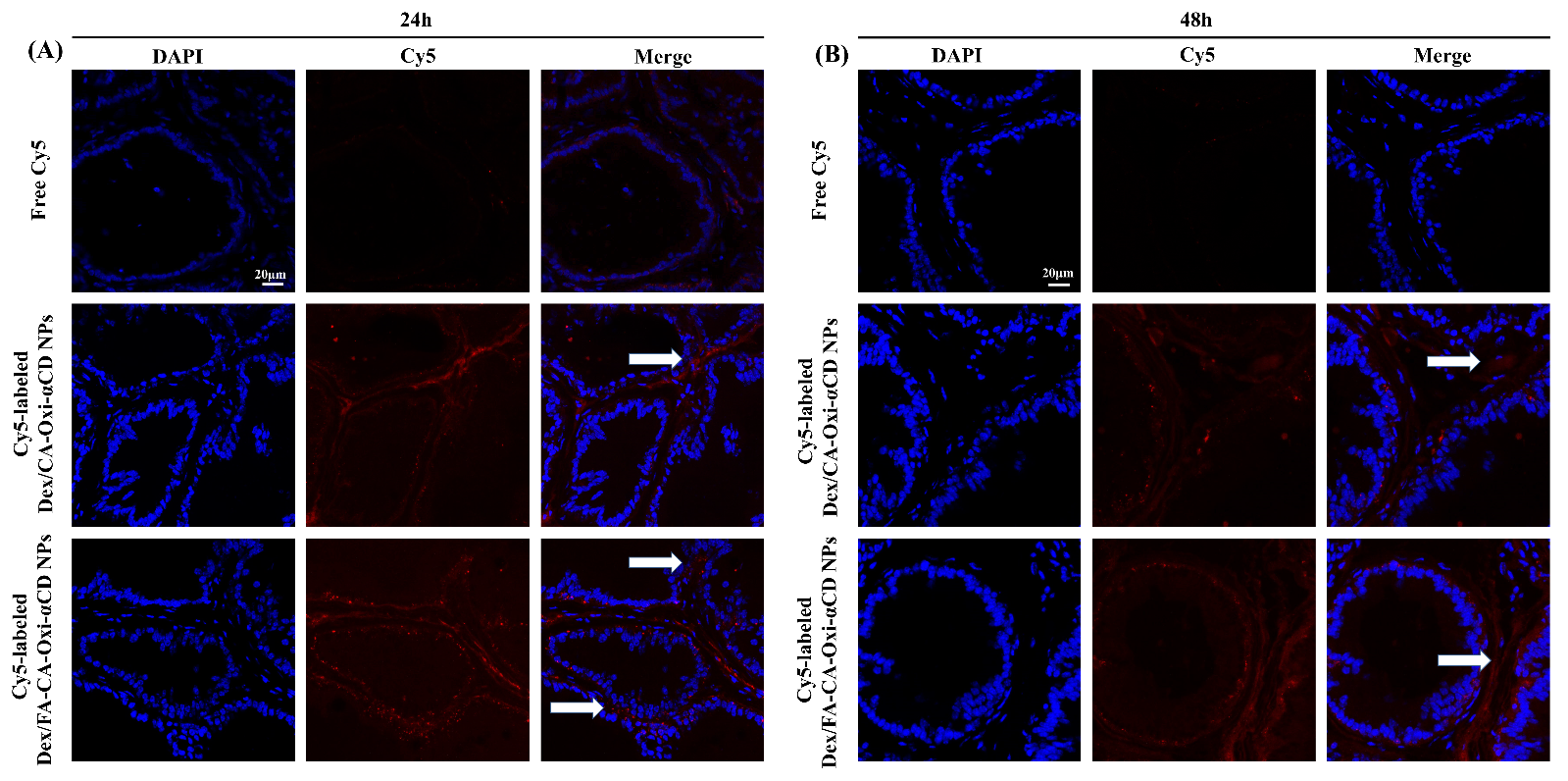
Figure S4. CLSM images of prostate tissues treated with Cy5 or Cy5-labeled NPs for 24 and 48 h. Red indicates the NPs, and blue indicates DAPI. The white arrow shows the accumulation of Cy5-labeled NPs in the glandular lumens. The scale bar represents 20 μm.


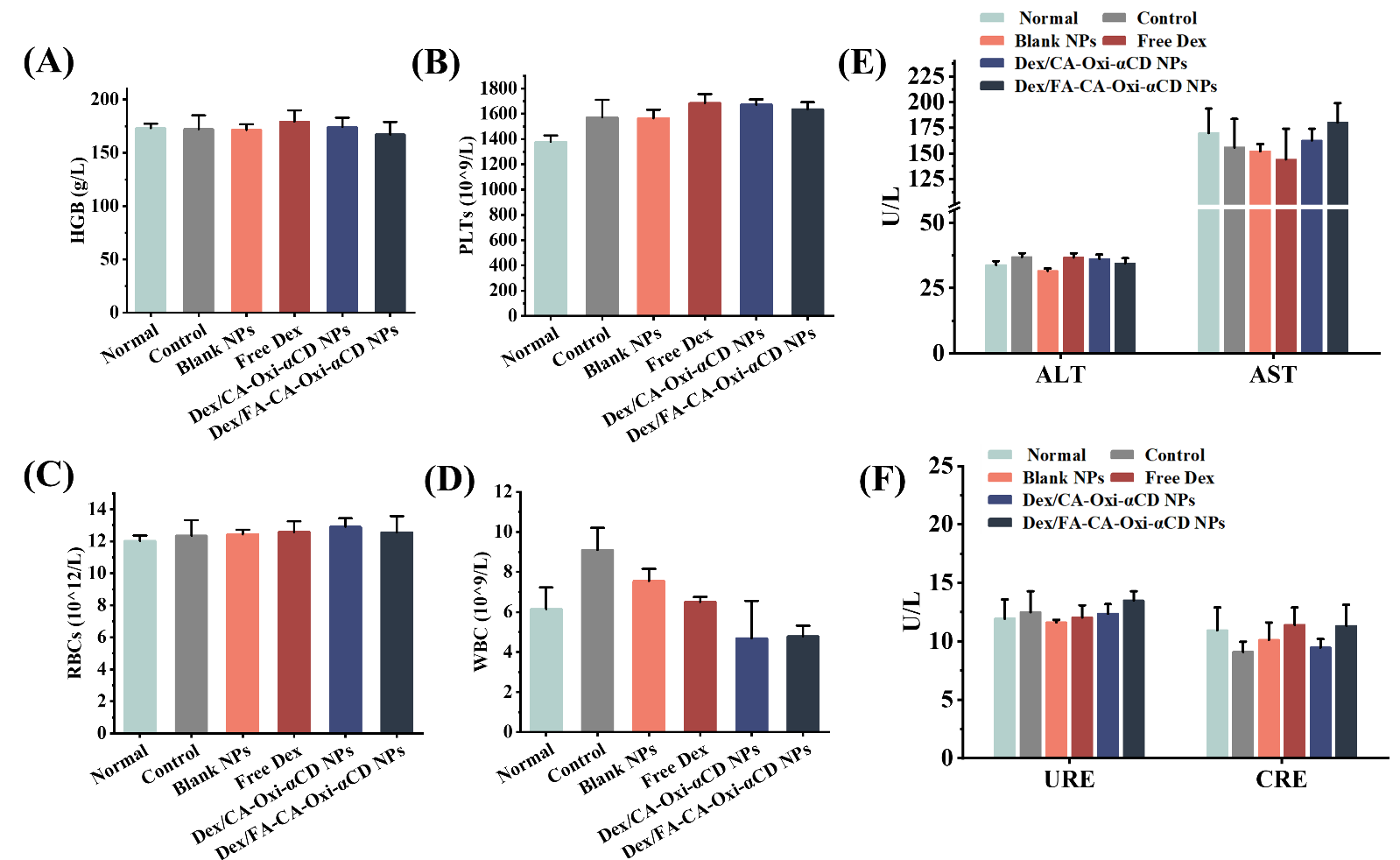


Figure S5. (A-D) Hematological parameters of blood samples from mice subjected to various treatments. WBC, white blood cell; HGB, hemoglobin; RBC, red blood cell; and PLT, platelet. (E) The levels of two biochemical markers relevant to liver functions. ALT, alanine aminotransferase; and AST, aspartate aminotransferase. (F) The concentration of markers related to renal function. CREA, creatinine; and UREA, urea.


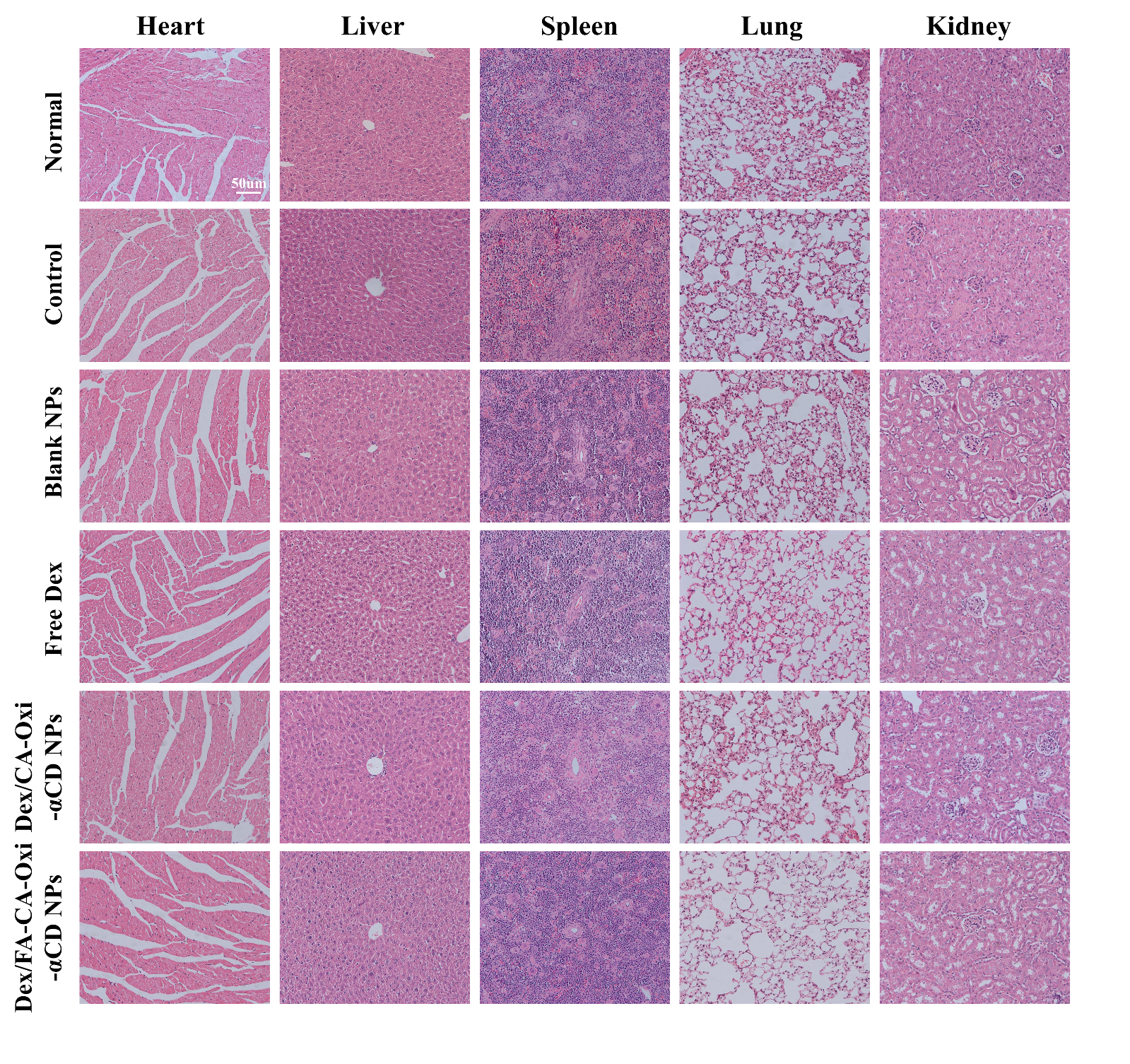


Figure S6. H&E-stained sections of the heart, liver, spleen, lung, and kidney from normal and EAP mice treated with saline, blank NPs, free Dex, Dex/CA-Oxi-αCD NPs, and Dex/FA-CA-Oxi-αCD NPs. The scale bar represents 50 μm.
